# γ-Aminobutyric Acid (GABA) Priming Improves Seed Germination and Seedling Stress Tolerance Associated With Enhanced Antioxidant Metabolism, *DREB* Expression, and Dehydrin Accumulation in White Clover Under Water Stress

**DOI:** 10.3389/fpls.2021.776939

**Published:** 2021-12-03

**Authors:** Min Zhou, Muhammad Jawad Hassan, Yan Peng, Lin Liu, Wei Liu, Yan Zhang, Zhou Li

**Affiliations:** College of Grassland Science and Technology, Sichuan Agricultural University, Chengdu, China

**Keywords:** ascorbic acid-glutathione cycle, oxidative damage, osmotic adjustment, transcription factor, gene expression

## Abstract

As an important plant growth regulator, the role of γ-aminobutyric acid (GABA) in regulating seeds germination was less well elucidated under water stress. The present study was conducted to investigate the impact of GABA pretreatment on seeds germination of white clover (*Trifolium repens*) under water deficient condition. Results demonstrated that seeds pretreated with 2μmol/l GABA significantly alleviated decreases in endogenous GABA content, germination percentage, germination potential, germination index, root length, and fresh weight along with marked reduction in mean germination time after 7days of germination under drought stress. In addition, seeds priming with GABA significantly increased the accumulation of soluble sugars, non-enzymatic antioxidants [reduced ascorbate, dehydroascorbic acid, oxidized glutathione (GSSG), and reduced glutathione (GSH)], and enzymes [superoxide dismutase (SOD), peroxidase (POD), catalase (CAT), ascorbate peroxidase (APX), dehydroascorbate reductase (DHAR), glutathioe reductase, and monodehydroasorbate reductase (MDHR)] activities involved in antioxidant metabolism, which could be associated with significant reduction in osmotic potential and the accumulation of superoxide anion, hydrogen peroxide, electrical leakage, and malondialdehyde in seeds under drought stress. The GABA-pretreated seeds exhibited significantly higher abundance of dehydrin (DHN, 56 KDa) and expression levels of DHNs encoding genes (*SK2*, *Y2K*, *Y2SK*, and *Dehydrin b*) and transcription factors (*DREB2*, *DREB3*, *DREB4*, and *DREB5*) than the untreated seeds during germination under water-limited condition. These results indicated that the GABA regulated improvement in seeds germination associated with enhancement in osmotic adjustment, antioxidant metabolism, and *DREB*-related *DHNs* expression. Current study will provide a better insight about the GABA-regulated defense mechanism during seeds germination under water-limited condition.

## Introduction

More than one third of the earth’s land is distributed in arid and semi-arid areas worldwide. In China, the arid area is about 3.32 million square kilometers, accounting for 34.6% of the total land area (Wang et al., 2012). Due to the increased anthropogenic activities and climate change, drought has become a critical problem limiting agricultural productivity worldwide (Li and Qiu, 2003). Seeds germination is highly sensitivity to drought stress because water is a crucial factor during germination (Liu et al., 2016). It has been reported that drought or osmotic stress decreased germination percentage, root length, fresh weight, seed vigor index (SVI), and prolonged mean germination time (MGT) in many economically important crop seeds (Okçu et al., 2005; Sun et al., 2006; Zhu et al., 2006; Sheteiwy et al., 2018). Seeds also undergo a series of physiological and biochemical changes, such as a decrease in osmotic potential (OP), accelerated oxidative damage, enhanced antioxidant metabolism, and other metabolic pathways in response to water stress or other stress (He et al., 2017; Cao et al., 2018). Under normal condition, the production and cleansing of reactive oxygen species (ROS) are in a dynamic balance. However, water stresses such as drought promote the production of ROS leading toward cell membrane lipid peroxidation, thus inhibiting seeds germination and seedling growth (Lyons and Raison, 1970; Na et al., 2012). To resist this threat, plants are inclined to enhance activities of various antioxidant enzymes, including superoxide dismutase (SOD), peroxidase (POD), ascorbate peroxidase (APX), and catalase (CAT) and promote non-enzymatic antioxidants metabolism, such as reduced glutathione (GSH), oxidized glutathione (GSSG), ascorbic acid (ASA), and dehydroascorbic acid (DHA). The study of Wang et al. (2009) had found that the stress-tolerant alfalfa (*Medicago sativa*) “Xinmu No.1” exhibited significantly lower ROS accumulation and lipid peroxidation associated with higher SOD, POD, APX, and CAT activities than the stress-sensitive “Northstar” during seed germination under drought stress. Similar findings were also found in other plant species during seeds germination under other stresses (Li et al., 2017). Therefore, the maintenance of better antioxidative capacity is essential for seeds germination when seeds are subjected to an unfavorable environmental condition.

For a long time, seed priming with water or chemicals has been used as an effective technique to improve seeds germination under favorable condition or enhance stress tolerance under environmental stress (Harris et al., 2001). Previous studies found that seed priming confers the tolerance to different abiotic stresses associated with alterations in physiological, molecular, and metabolic levels during seeds germination. For example, seed priming with spermidine and 5-aminolevulinic acid improved amylolysis, antioxidant defense, and polyamines metabolism during rice (*Oryza sativa*) seeds germination under chilling stress (Sheteiwy et al., 2017). Enhanced starch metabolism and antioxidant capacity induced by the spermidine priming were observed during white clover (*Trifolium repens*) seeds germination under water stress (Li et al., 2014). Polyethylene glycol (PEG)-induced osmotic stress could be effectively alleviated by priming with methyl jasmonate through regulating metabolic profile in rice seeds (Sheteiwy et al., 2018). In addition, seed priming with γ-aminobutyric acid (GABA) could significantly alleviate salt-induced inhibition of seeds germination associated with changes in physiological, metabolic, and molecular responses in white clover (Cheng et al., 2018). It is worth further exploring effect and mechanism of seed priming with different chemicals on alleviating stress damage under various abiotic stresses. The GABA is an important non-protein amino acid that exists naturally in animals and plants (Kinnersley and Turano, 2000). Generally, the plant tissues contain minute concentration of GABA under normal conditions, but it can be amplified in different plant species under various stressed conditions, such as in soybean (*Glycine max*) leaves under lower temperature (Wallace et al., 1984), in creeping bentgrass (*Agrostis stolonifera*) under drought and heat stress (Li et al., 2019a, 2020), and in white clover under drought stress (Yong et al., 2017). Many studies have also found that GABA played a vital role in the stress tolerance of plants associated with the regulation of the tricarboxylic acid cycle, nitrogen reservoir, cytoplasmic pH, antioxidant defense, and osmotic potential (Guo-Xing et al., 2003; Khan et al., 2021; Li et al., 2021). Exogenous GABA application could improve the hypoxic tolerance in muskmelon (*Cucumis melo*; Fan, 2012) and cucumber (*Cucumis sativus*; Huang, 2016) seedlings. In addition, the GABA application significantly increased the activity of POD and APX and the accumulation of osmolytes, thus effectively alleviating the oxidative damage and water imbalance in the leaves of perennial ryegrass (*Lolium perenne*) under water deficient condition (Krishnan et al., 2013). The GABA enhanced antioxidant metabolism to mitigate oxidative damage, which is a key regulatory pathway for improving drought tolerance of creeping bentgrass (Tang et al., 2020). Although the GABA is beneficial for plants adaption to abiotic stresses, the study about its function during seeds germination is still at initial stages.

Large number of late embryonic development proteins, also known as dehydrins (DHNs), tend to accumulate during seeds germination under stress conditions (Houde et al., 1995; Cheng et al., 2018). It has been reported that DHNs are involved in the water regulation, biomembrane protection, and antioxidant defense, hence contributing toward adaptation to drought stress in plants (Dure, 1992; Roberts et al., 1993; Hara, 2009). Baldwin et al. (1999) found that wheat (*Triticum aestivum*) embryos accumulated abundant DHNs under osmotic stress. The content of DHNs in drought-tolerant soybean (*Glycine max*) varieties was higher than that in drought-sensitive soybean varieties in response to drought stress (Arumingtyas et al., 2013). Na^+^ priming mitigated the inhibition of seeds germination associated with the upregulation of a *DHN* gene *SK2* in white clover under drought stress (Cao et al., 2018). Transcript level of *DHNs* is regulated by multiple transcription factors, including MYC/MYB, bZIP, and DREB family. Many *DHNs* include the DREB recognition sequence “dehydration-responsive element (DRE)” in the promoter that can recognize and combine DREBs (Maria et al., 2017). Previous research has already confirmed that significant increase in *DREB/CBF* expression level through transgenic approach could enhance the expression of downstream target genes encoding DHNs (Battaglia et al., 2008). However, it is still unclear whether specific *DREBs* and *DHNs* genes are regulated by GABA during seeds germination under water-limited condition.

White clover is an important perennial legume and cultivated all over the world (Li et al., 2012), due to its soft stems, abundant leaves, and high nutritional value, thus utilizing as an excellent feed source for livestock. White clover is also used as an imperative ground cover plant in urban areas because of its fast regeneration ability and high ornamental value (Sincik and Acikgoz, 2007). However, white clover mainly adapts to warm and moist climate, and water deficit easily affects seeds germination, forage quality, and ornamental value. Objectives of this study were (1) to investigate seeds germination characteristics, antioxidant defense system, and osmotic responses regulated by the GABA and (2) to further reveal *DREBs* expression and DHNs accumulation associated with the GABA-induced drought tolerance, which will provide important information for better understanding of the GABA-regulated mechanisms of drought tolerance in plants during seeds germination.

## Materials and Methods

### Plant Materials and Treatments

White clover seeds (cultivar “Haifa”) were used as test materials. For GABA pretreatment, the seeds were soaked in distilled water for 1h, and then drenched in 2μmol/l GABA solution for another 2h, while untreated seeds were soaked in deionized water for 3h in the dark. After the surface moisture of seeds being removed, 50 seeds were randomly selected and placed in petri dishes containing 10ml distilled water or polyethylene glycol-6000 (PEG-6000, −0.3 Mpa) solution with three layers of filter papers. All petri dishes were kept in a growth chamber at 23/19°C (day/night) with 12h photoperiod. The experimental design was a completely randomized block design with two water status (normal water condition and water stress condition) and the GABA application under each water status. Four treatments were set: (1) water (seeds germination in distilled water); (2) water+GABA (seeds primed with GABA and then germination in distilled water); (3) PEG (seeds germination in PEG-6000 solution); and (4) PEG+GABA (seeds primed with GABA and then germination in PEG-6000 solution). Each treatment included eight independent replicates. Petri dishes, distilled water, and PEG-6000 solution were refreshed every day. Four replicates were selected from each treatment on the 3rd and 7th day of germination to measure germination characteristics and physiological and biochemical parameters of germinated seeds. The effective concentration of GABA (2μM) was applied in this study based on a preliminary test with a range of concentrations (0.5, 1.0, 2.0, 4.0, and 8.0μM), since 2μM GABA exhibited the most pronounced effect on alleviating germination inhibition.

### Determination of Seed Germination Characteristics

The germination percentage (GP) was measured on the 7th day of seed germination: GP (%)=*n*/*N*×100, where *N* is the total number of seeds, and *n* is the number of seeds that have germinated after 7days interval. The germination vigor (GV, %) was calculated as a percentage of germinated seeds on the 3rd day of germination. The germination index (GI) was calculated according to the formula ∑(*Gt*/*Dt*), where *Gt* is the number of germinated seeds, and *Dt* is the corresponding time to *Gt* in days. The formula for MGT=∑(*D*×*n*)/∑*n* was used for calculating the MGT, where *D* is the number of days, and *N* is the number of germinations in the corresponding days. On the 7th day of germination, 10seedlings were randomly selected from each replicate to measure their root length, fresh weight (FW), and dry weight (DW). The SVI was calculated by following formula: VI=FW×GI.

### Determination of Osmotic Potential, Soluble Sugar, and Endogenous GABA Content

On the 7th day of germination, fresh samples (0.2g) were taken to measure OP according to the method of Blum (1989). The samples were soaked in distilled water at 4°C for 8h and tissue blotted to remove surface water. The samples were pressed to get cell saps, and then the osmotic pressure (c) of cell saps was measured using an osmotic pressure meter. The OP was converted according to the formula (Mpa=−C×2.58×10^−3^). The soluble sugar content was measured using 0.2g of dry seedlings samples after 7days of germination following the protocol of Li et al. (2015). On the 7th day of germination, 0.1g of fresh samples were taken to determine endogenous GABA content by using ELISA Kit. The Assay Kit (Art. No. G1106F) was purchased from Shanghai Enzyme-linked Biotechnology Co., Ltd., China.

### Determination of Oxidative Damage and Antioxidant Metabolism

The superoxide anion ( $O_{2}^{\cdot -}$ ) was measured following the method of Elstner and Heupel (1976), and hydrogen peroxide (H_2_O_2_) content was determined according to the procedures of Uchida et al. (2002). Electrolyte leakage (EL) was measured by using a conductivity meter (Blum and Ebercon, 1981), and the calculation formula was as follow EL=(the conductivity before cooking/the conductivity of killed tissues)×100%. In order to estimate the MDA and antioxidant enzyme activities, fresh samples were taken and 1.5ml precooled phosphoric acid buffer was added. Then, the mixture was ground mechanically and centrifuged for 20min at 12,000g and 4°C. The supernatant was collected and used for the determination of POD, SOD, APX, CAT, glutathione reductase (GR), dehydroascorbate reductase (DHAR), monodehydroasorbate reductase (MDHR), malondialdehyde (MDA), and soluble protein. The SOD was determined following the nitrogen blue tetrazole (NBT) method at 560nm (Ries, 1977). Changes of absorbance every 10s at 470nm were obtained for determination of the POD or CAT activity (Chance and Maehly, 1955). The APX, GR, DHAR, and MDHR were measured according to Nakano and Asada (1981) and Cakmak et al. (1993) at 240, 290, 340, 265, and 340nm by recording the change of absorbance every 10s. The MDA content was determined according to the method of Dhindsa et al. (1981) by adding reaction solution with 20% (*w/v*) trichloroacetic acid and 0.5% (*w/v*) thiobarbital into extraction solution, and heating in 95°C water bath for 15min, and then cooling rapidly in ice water bath. The absorbance of the supernatant was measured at 532 and 600nm after centrifugation for 10min at 12,000*g*. Soluble protein content was determined by using the coomassie bright blue method (Bradford, 1976). The GSH, GSSG, ASA, and DHA content were detected by using the Assay Kit purchased from Suzhou Grace Biotechnolgy Co., Ltd., China. (Art. No. G0602F, Art. No. G0207F, Art. No. G0201F, and Art. No. G0202W).

### Determination of Dehydrin Accumulation

The abundance of DHNs was measured using Western blot analysis. About 0.3g of fresh samples were collected and utilized for extracting the protein. Details about assay method have been described in our previous study (Li et al., 2015).

### Genes Expression Analysis

On the 3rd and 7th day of germination, 0.1g fresh samples were taken, and four biological replicates and one technical replicate were used for extraction RNA. Total RNA was extracted from fresh samples using a total RNA extraction kit (Qiagen). Later, the extracted RNA was reverse-transcripted into cDNA using a reverse transcription kit (Fermentas). Finally, primers of tested genes (Table 1) were used for amplification under real-time quantitative fluorescence PCR (qRT-PCR). The PCR procedure for all genes was: 94°C for 5min, denaturation at 95°C for 30s (40 repeats), annealing at 58–60°C (Table 1) for 30s, and extension at 72°C for 30s.

**Table 1:** Primer sequences and corresponding GeneBank accession numbers of the analyzed genes.

Targetgene	Accession No.	Forward Primer (5'-3')	Reverse Primer (5'-3')	Tm (°C)
*SK2*	GU443960.1	TGGAACAGGAGTAACAACAGGTGGA	TGCCAGTTGAGAAAGTTGAGGTTGT	58
*Y2K*	JF748410.1	AGCCACGCAACAAGGTTCTAA	TTGAGGATACGGGATGGGTG	60
*Y2SK*	GU443965.1	GTGCGATGGAGATGCTGTTTG	CCTAATCCAACTTCAGGTTCAGC	60
*Dehydrin b*	GU443960.1	TCCAGTCATCCAGCCTGTTG	CCAGCCACAACACTTGTCA	60
*DREB2*	EU846194.1	CAAGAACAAGATGATGATGGTGAAC	AAGAAGAAGAATTGGAGGAGTCATG	58
*DREB3*	EU846196.1	GCTCAATAGGACTCAACCAACTCAC	TGACGTTGTCTAACTCCACGGTAA	58
*DREB4*	EU846198.1	CTTGGTTGTGGAGATAATGGAGC	AAGTTGCAATCTGAATTCTGAGGAC	58
*DREB5*	EU846200.1	GCGATAGGTTCAAAGAAAGGGTG	AGAGCAGCATCTTGAGCAGTAGG	58
*β-Actin*	JF968419	TTACAATGAATTGCGTGTTG	AGAGGACAGCCTGAATGG	58

### Statistical Analysis

The data were analyzed by using Microsoft Excel 2016, and the variance analysis and single factor significance relationships were tested with SPSS 26.0 (IBM, Armonk, NY, United States) at *p*≤0.05.

## Results

### Effects of GABA Priming on Germination Characteristics and Endogenous GABA Content During Seed Germination Under Normal Condition and Water Stress

**Figure 1:**
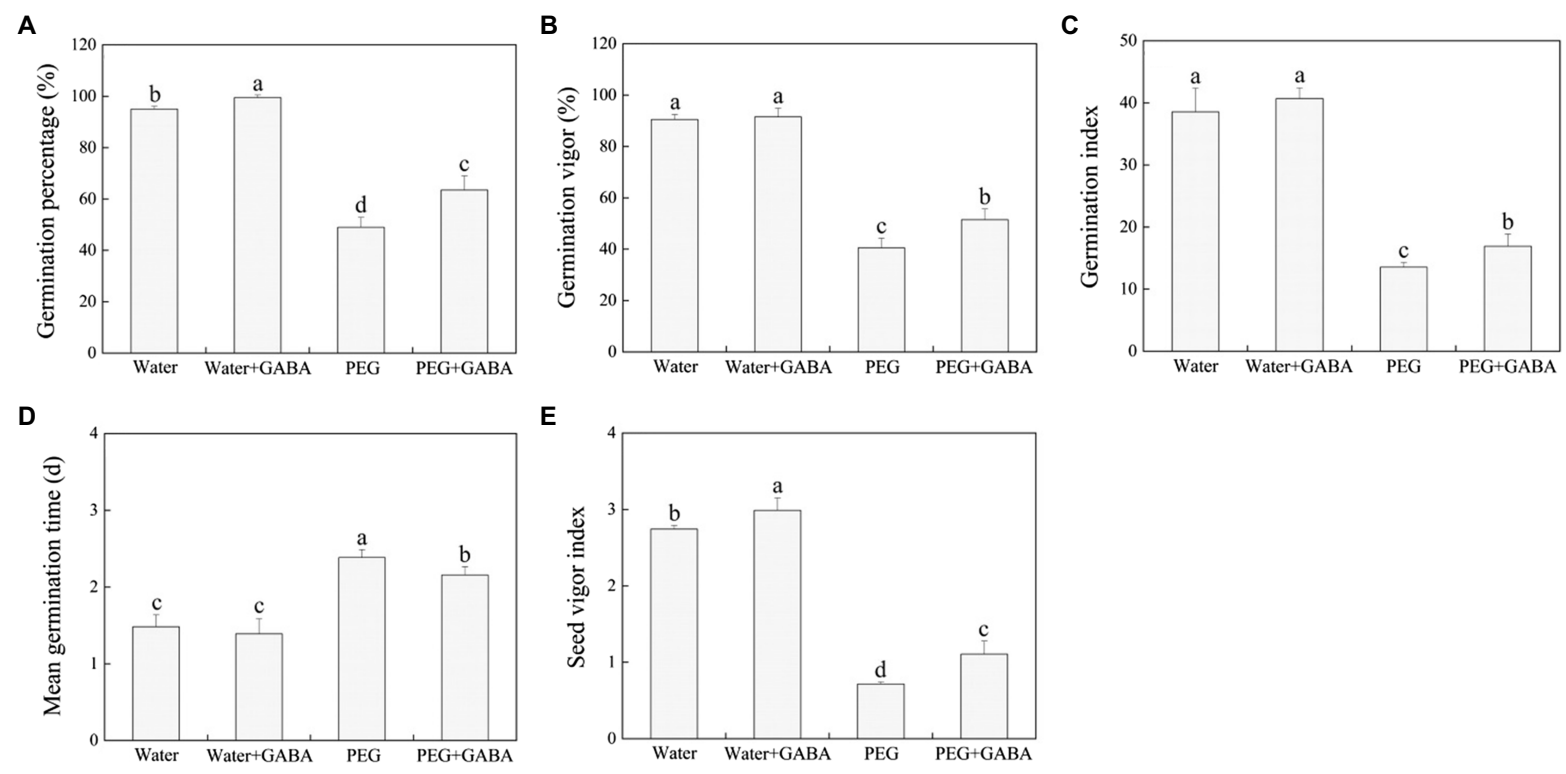
Effects of seed priming with deionized water or γ-aminobutyric acid (GABA) on germination characteristics [**(A)** germination percentage (GP), **(B)** germination vigor (GV), **(C)** germination index (GI), **(D)** mean germination time (MGT), and **(E)** seed vigor index (SVI)] of white clover seeds under water stress.

Water stress significantly decreased GP, GV, GI, and SVI, while the MGT significantly increased under water-limited condition (Figure 1). Under normal water condition, exogenous GABA exhibited no significant effects on GV, GI, and MGT, but exogenous application of GABA significantly increased GV and GI, and also reduced the MGT under water stress (Figures 1B–D). In addition, the GABA-primed seeds demonstrated significantly higher GP and SVI than untreated seeds under normal condition or water stress (Figures 1A,E).

**Figure 2:**
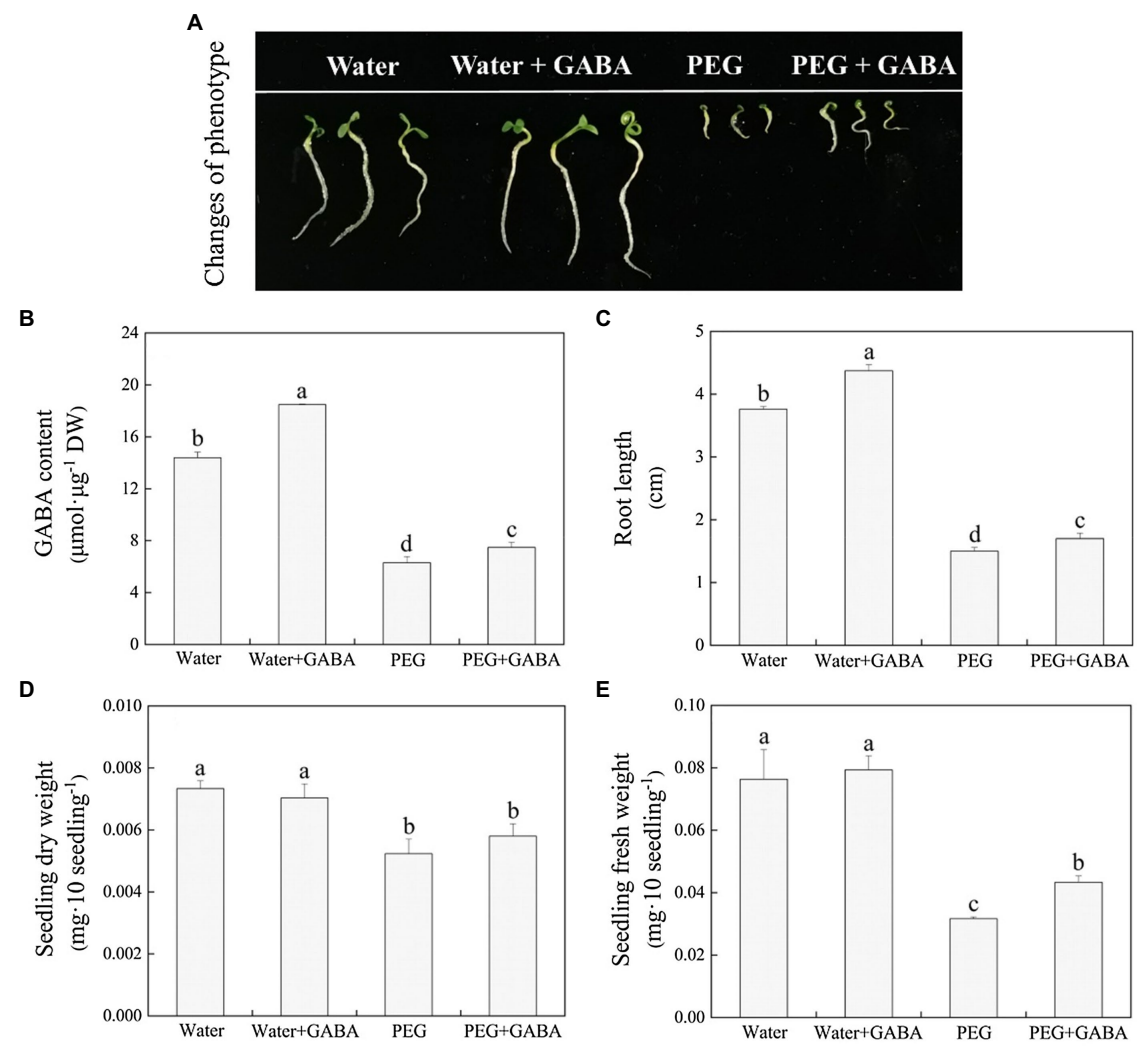
Effects of seed priming with deionized water or γ-aminobutyric acid (GABA) on **(A)** phenotypic change, **(B)** endogenous GABA content, **(C)** root length, **(D)** seedlings dry weight, and **(E)** seedlings fresh weight on the 7th day of germination in white clover under water stress.

Figure 2A showed the phenotypic differences among different treatments on 7th day of germination under normal condition and water stress. On the 7th day of germination, the endogenous GABA content in seedlings under normal condition and water stress was significantly different between the seeds primed with or without GABA (Figure 2B). The GABA-primed seeds exhibited 28.57 or 18.77% higher endogenous GABA content than the non-priming seeds under normal condition or water stress, respectively (Figure 2B). Water stress significantly inhibited root length of both GABA-primed and non-primed seedlings, but the seedlings pretreated with GABA showed significantly longer root length than non-primed seeds after 7days of germination under normal and water stress conditions (Figure 2C). Seedlings DW and FW declined significantly under water stress (Figures 2D,E). The GABA priming did not show significantly effect on seedlings DW under normal and water stress conditions (Figure 2D), but significantly improved seedlings FW under water stress (Figure 2E). These results showed that exogenous application of GABA could significantly improve endogenous GABA content and germination characteristics of white clover seeds under water stress.

### Effects of GABA Priming on Osmotic Potential and Soluble Sugar Content During Seed Germination Under Normal Condition and Water Stress

**Figure 3:**
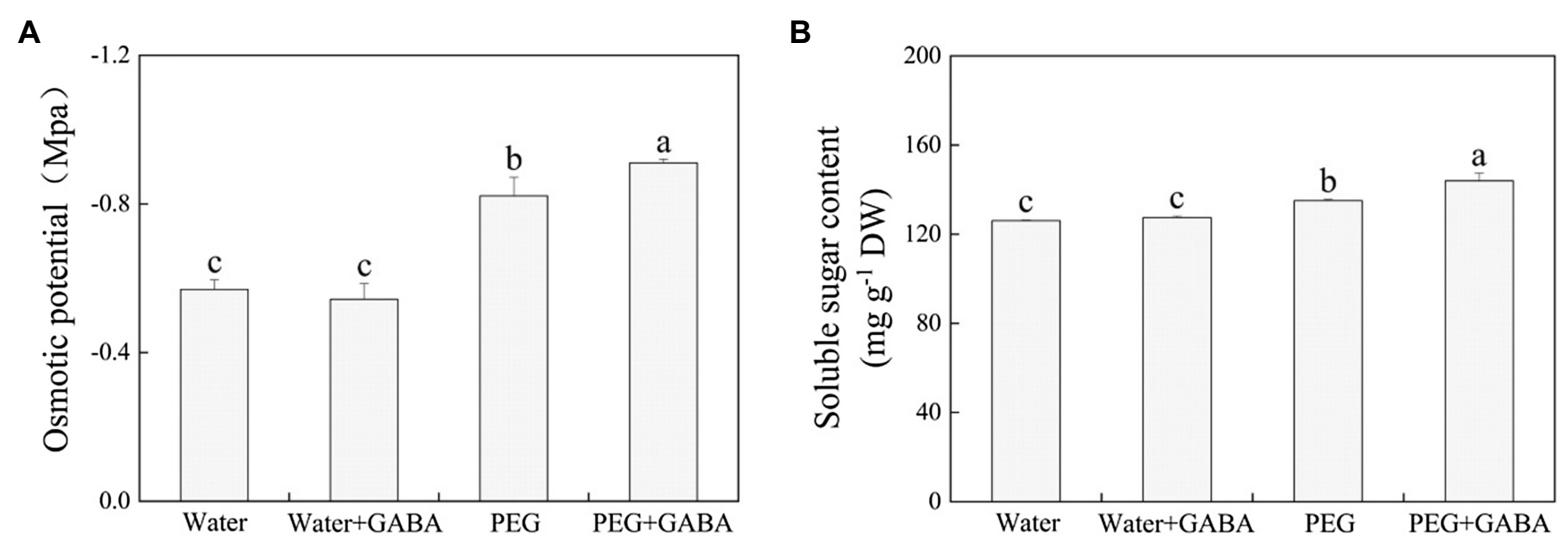
Effects of seed priming with deionized water or γ-aminobutyric acid (GABA) on osmotic potential (OP; **A**) and soluble sugar content **(B)** during seed germination of white clove under water stress.

On the 7th day of germination, seeds priming with or without GABA showed no significant effect on OP and soluble sugar under normal condition (Figure 3). Water stress induced a significant decline in OP with marked increase in soluble sugars content in the GABA-primed or non-primed seedlings (Figures 3A,B). However, GABA pretreated seedlings showed significantly lower OP and higher soluble sugar content than seeds without GABA priming under water deficient conditions (Figures 3A,B). The GABA priming could significantly reduce the OP and also promote the accumulation of soluble sugar content in seedlings under water stress.

### Effects of GABA Priming on Oxidative Damage and Antioxidant Metabolism During Seed Germination Under Normal Condition and Water Stress

**Figure 5:**
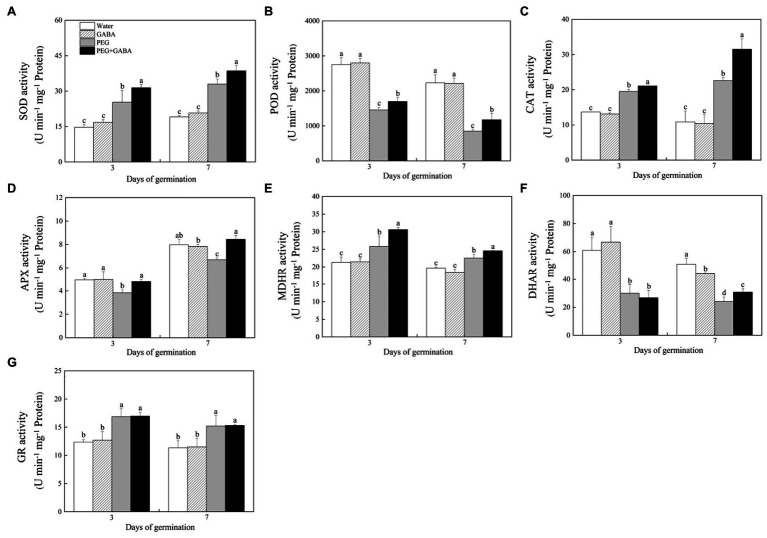
Effects of seed priming with deionized water or γ-aminobutyric acid (GABA) on the activities of superoxide dismutase (SOD; **A**), peroxidase (POD; **B**), catalase (CAT; **C**) and key ascorbate-glutathione cycle enzymes ascorbate peroxidase (APX; **D**), monodehydroasorbate reductase (MDHR; **E**), dehydroascorbate reductase (DHAR; **F**), glutathione reductase (GR; **G**) during the germination of white clover seeds under water stress.

On the 3rd and 7th day of germination, the GABA priming demonstrated no significant effects on the $O_{2}^{\cdot -}$ , H_2_O_2_, MDA content, and EL in seedlings under normal condition (Figures 4A–D). Under water stress, $O_{2}^{\cdot -}$ , H_2_O_2_, and MDA increased significantly in both GABA-primed and non-primed seedlings when compared to control (Figures 4A–C). However, the GABA priming significantly alleviated the oxidative damage during seed germination under water stress (Figures 4A–C). The EL significantly increased in the non-priming seeds on the 3rd and 7th day of water stress, but did not exhibit any significant differences between the GABA-primed seedlings under water stress and the control (Figure 4D). Under normal water condition, the GABA pretreatment had no significant effects on SOD, POD, and CAT activities during seeds germination (Figure 5). Compared with normal treatments (the “Water” and the “Water+GABA”), SOD and CAT activities were significantly increased in the “PEG” and “PEG+GABA” treatments, however, the highest values for activities of these two enzymes were observed in “PEG+GABA” treatment (Figures 5A,C). On the 3rd and 7th day of seeds germination, the POD activity significantly decreased under water stress, whereas GABA priming significantly alleviated the decrease in SOD activity (Figure 5B).

**Figure 4:** Effects of seed priming with deionized water or γ-aminobutyric acid (GABA) on superoxide anion (O_2_^−^; **A**), hydrogen peroxide (H_2_O_2_; **B**), electrical leakage (EL; **C**) and malondialdehyde (MDA; **D**) during seed germination of white clover under water stress.

As shown in Figure 5D, osmotic stress significantly inhibited APX activity in seedlings without the GABA pretreatment on 3rd and 7th day of germination. In GABA-pretreated seedlings, the APX activity was not significantly affected by water stress (Figure 5D). The DHAR activity significantly declined, but MDHR and GR activities were significantly increased during seeds germination under water stress (Figures 5E–G). The MDHR activity was significantly enhanced by GABA pretreatment on the 3rd and 7th day of germination under water stress (Figure 5E). However, seeds priming with the GABA had no significant effect on the DHAR and GR activities on the 3rd or 7th day of germination (Figures 5F,G).

**Figure 6:**
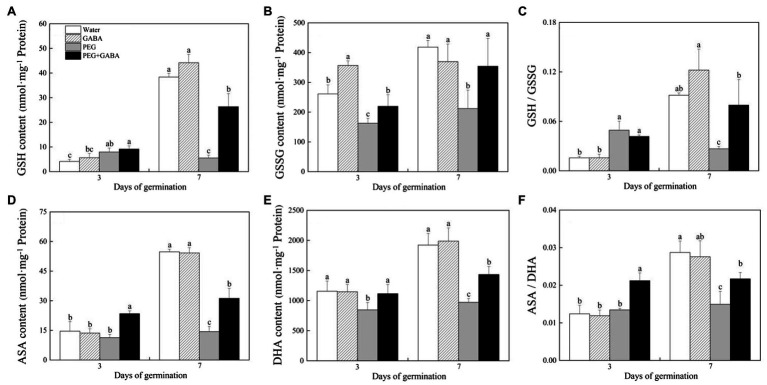
Effects of seed priming with deionized water or γ-aminobutyric acid (GABA) on content of non-enzymatic antioxidants glutathione (GSH; **A**), reduced glutathione (GSSG; **B**), the GSH/GSSG ratio **(C)**, ascorbic acid (AsA; **D**), dehydroascorbic acid (DHA; **E**), and the ASA/DHA ratio **(F)** during the germination of white clover seeds under water stress.

Water stress induced significant increase in GSH contents in all seedlings, but no significant difference was detected between the “PEG” and the “PEG+GABA” on the third day of germination (Figure 6A). The GABA-pretreated seeds exhibited significantly higher the GSSG content than the non-pretreated seeds on the 3rd day of germination (Figure 6B). On the 7th day of germination, water stress significantly reduced the GS, GSSG, and the ratio of GSH/GSSG in seedlings without the GABA pretreatment, however, the GABA-pretreated seeds had a 78.95, 40.15, or 66.46 increase in GSH, GSSG, or GSH/GSSG than the seeds without GABA pretreatment on the 7th day of germination, respectively (Figures 6A–C). The GABA-pretreated seeds also maintained significantly higher the ASA, DHA, and ASA/DHA than the seeds without GABA pretreatment on the 3rd day of germination (Figures 6D–F). In addition, water stress significantly decreased the ASA, DHA, and ASA/DHA in all seedlings on the 7th day of germination, whereas the GABA-pretreated seeds exhibited 53.94, 32.30, and 31.17% increase in ASA, DHA, or ASA/DHA than the seeds without GABA pretreatment on the 7th of germination, respectively. The GABA piming could significantly activate antioxidant defense and reduce oxidative damage during seeds germination under water stress.

### Effects of GABA Priming on Relative Expression Levels of DREBs and Dehydrins Genes During the Germination Under Normal Condition and Water Stress

**Figure 7:**
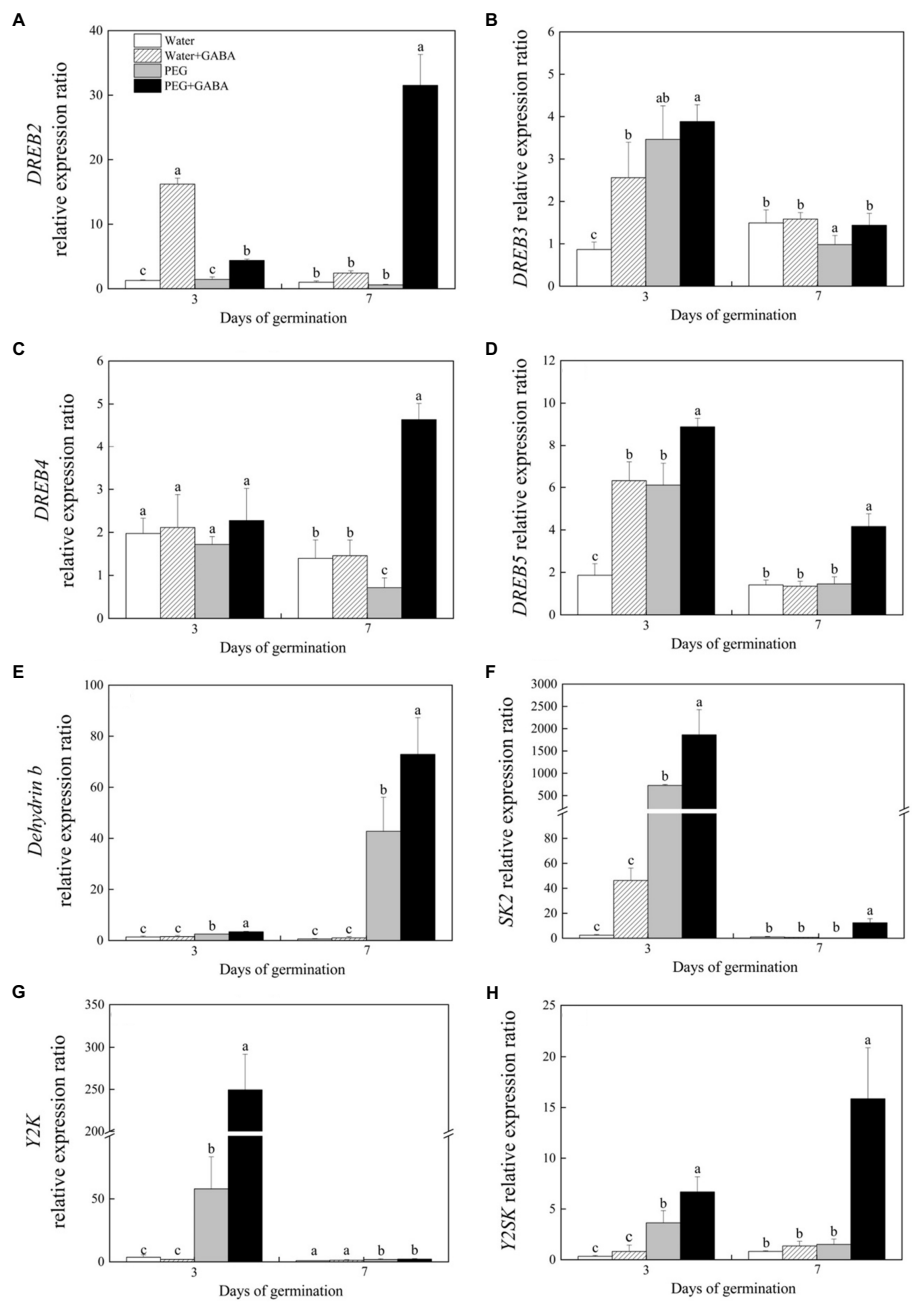
Effects of seed priming with deionized water or γ-aminobutyric acid (GABA) on the relative expression levels of *DREB2* **(A)**, *DREB3* **(B)**, *DREB4* **(C)**, and *DREB5* **(D)** transcription factors and the relative expression levels of *Dehydrin b* **(E)**, *SK2* **(F)**, *Y2K* **(G)**, and *Y2SK* **(H)** during the germination of white clover seeds under water stress.

On the 3rd day of germination, seeds priming with GABA showed significantly higher *DREB2* expression level than seeds without GABA priming under normal condition or water stress (Figure 7A). The *DREB2* expression level was not affected by water stress in the untreated seeds, but enormously increased in the treatment with GABA priming (Figure 7A). The GABA application demonstrated no significant effect on *DREB3* expression under water stress, but had significant effect on *DREB3* under normal condition on the 3rd day (Figure 7B). Under normal water condition, the *DREB4* expression level did not show significant differences among four treatments on the 3rd day of germination (Figure 7C). On the 7th day of germination, water stress significantly inhibited the *DREB3* and *DREB4* expression in seedlings without the GABA priming, but significantly upregulated *DREB3* and *DREB4* expression in the seedlings primed with GABA (Figures 7B,C). On the 3rd day of germination, water stress or GABA treatment under normal condition significantly upregulated the *DREB5* expression, and the PEG-stressed treatment primed with GABA (PEG+GABA) showed significantly higher *DREB5* expression than other treatments (Water, Water+GABA, and PEG) on the 7th day of germination (Figure 7D).

Under normal water condition, seeds priming with GABA had no significant effects on the transcription levels of *Dehydrin b*, *Y2K*, and *Y2SK* on the 3rd and 7th day of germination (Figures 7E,G,H). Water stress significantly increased the transcription level of *Dehydrin b* and the GABA priming further enhanced this effect during seeds germination (Figure 7E). On the 3rd day of germination, the GABA priming significantly increased the *SK2* expression under normal and water deficient conditions (Figure 7F). On the 7th day of germination, only the expression level of *SK2* was significantly increased in the GABA-priming treatment under water stress (Figure 7F). During seeds germination (on day 3 and 7), the *Y2K* transcription level significantly increased in response to water stress in GABA-primed and non-primed treatments, and the GABA-primed treatment showed 93.27% increase in the *Y2K* transcription level than the non-priming treatment on the 3rd day of germination under water stress, but there is no significant difference on the 7th day (Figure 7G). The GABA-pretreated seedlings exhibited 20.41 or 65.91% higher *Y2SK* expression than the untreated seedlings on 3rd or 7th day of germination under water stress, respectively (Figure 7H). In response to water stress, the GABA regulated seeds germination of white clover associated with *DREB* and dehydrin pathways.

### Effects of GABA Priming on Dehydrins Accumulation During Seed Germination Under Normal Condition and Water Stress

**Figure 8:**
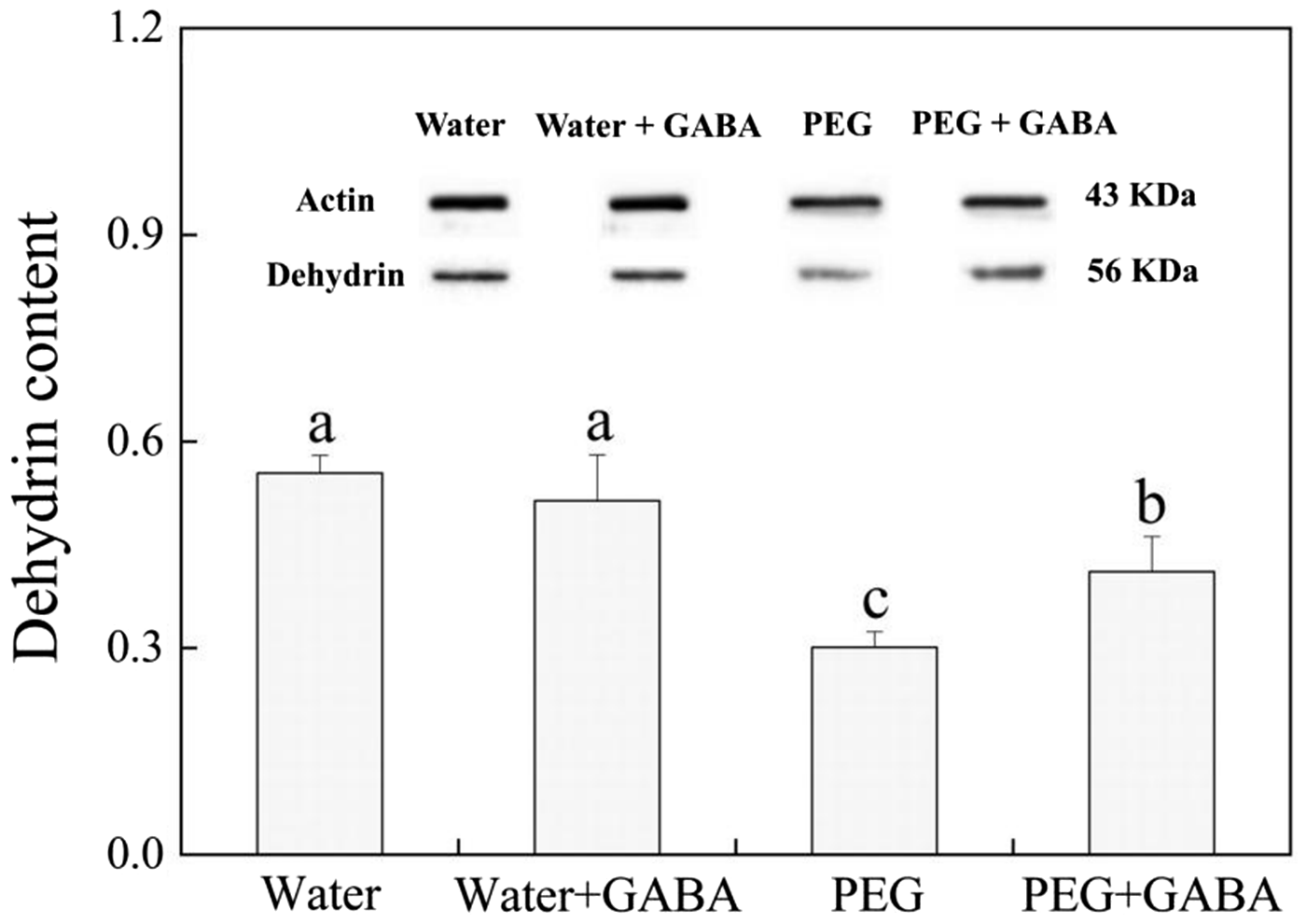
Effects of seed priming with deionized water or γ-aminobutyric acid (GABA) on the abundance of dehydrins on the 7th day of germination in white clover under water stress.

**Figure 9:**
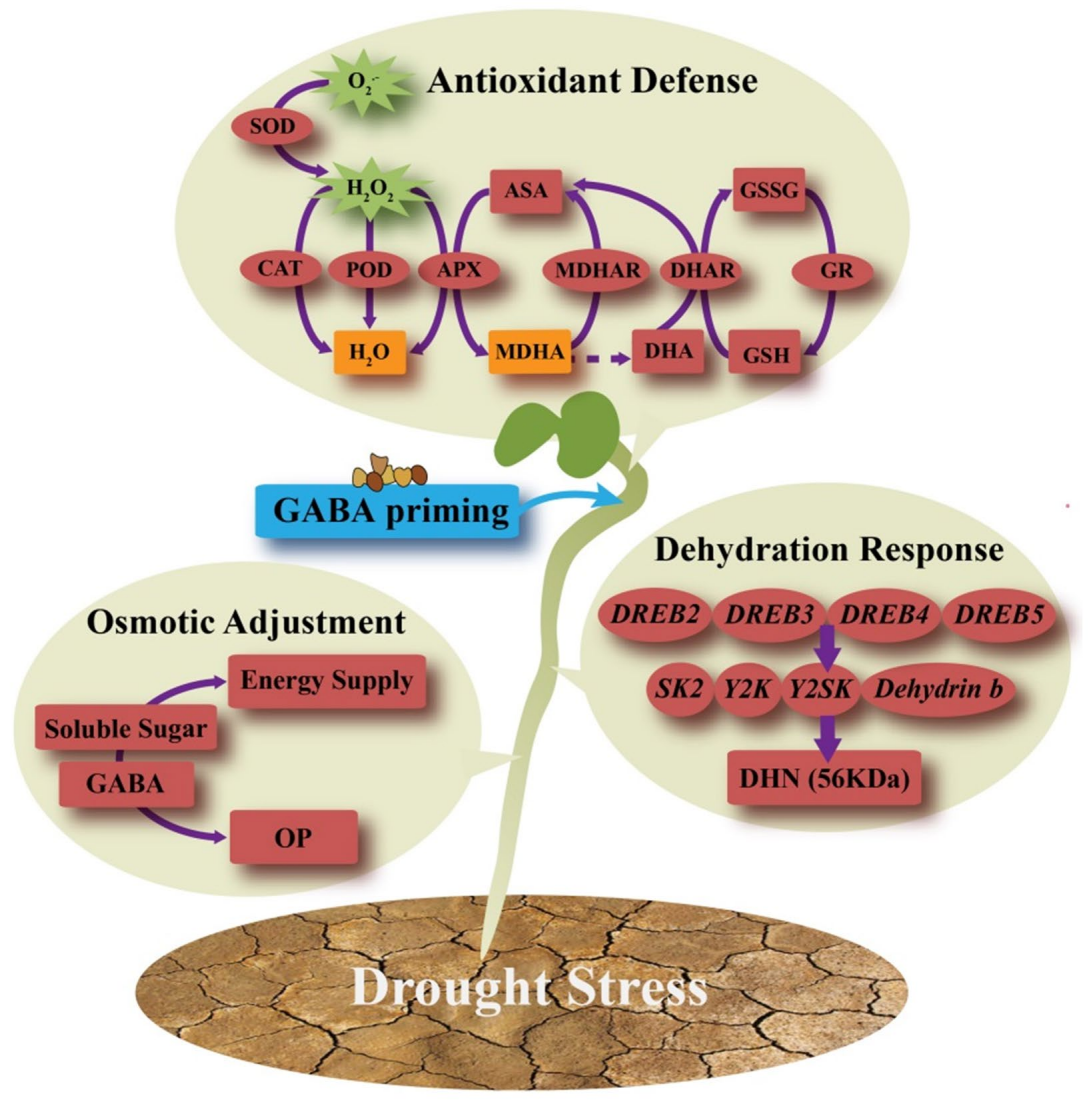
Integrative pathways were regulated by the γ-aminobutyric acid (GABA) priming during seeds germination of white clover.

As shown in Figure 8, the GABA priming had no significant effect on the DHN (56 KDa) content on the 7th day of germination under non-stress condition. Water stress significantly decreased the abundance of DHN in non-GABA priming treatment, and compare to that, the GABA-primed treatment showed 36.48% increase in the abundance of DHN under water stress (Figure 8). Figure 9 showed that integrative pathways were regulated by the GABA priming during seeds germination of white clover. The GABA-induced DHN (56 KDa) accumulation could be one of important regulatory mechanisms during seeds germination suffering water stress.

## Discussion

Seed germination is the most critical phase in plant’s life cycle as it determines plants growth and subsequent adaptation to various stress conditions. An earlier study has showed that the 15% PEG could significantly inhibit seeds germination of white clover, and with the increase in PEG concentration (18–20%), inhibitory effects further aggravated (Li et al., 2014). It was also found that the PEG significantly inhibited rice seed germination in the study of Sheteiwy et al. (2018). In addition, our previous study found that the GABA priming effectively alleviated declines in GP, GV, GI, and SVI associated with the maintenance of higher endogenous GABA content in GABA-pretreated white clover seedlings during germination under salt stress (Cheng et al., 2018). The current findings showed that water stress significantly inhibited seeds germination of white clover and decreased the SVI, FW, DW, and root length. However, the GABA priming significantly alleviated stress-induced decreases in endogenous GABA content, GV, GI, SVI, FW, and root length with substantial reduction in MGT during seeds germination of white clover under water stress (Figures 1, 2). These results indicate the beneficial role function of GABA during seeds germination under water stress.

The production and utilization of soluble sugars are the most essential processes during seeds germination, as they act as energy reservoirs for seeding growth. Being one of the most important osmolytes in plants, the accumulation of soluble sugars reduces OP in cells so that plants can maintain better water requirement and balance under water stress (Morgan, 1984). Previous studies have found that exogenous GABA treatment increased soluble sugars content and reduced the OP in cells of different plant species under various abiotic stresses, including flooding stress, drought, and salt stress (Gao et al., 2007; Sheteiwy et al., 2019). Significant decline in OP and increase in soluble sugars content were observed in seedlings of white clover after 7days of germination under water stress (Figure 3). Similar results were found in previous studies about soluble sugars accumulation during seeds germination of white clover or other plant species (Abdul et al., 2011; Cao et al., 2018). More importantly, the GABA priming further amplified the soluble sugars accumulation and also decreased the OP significantly when compared with untreated seedlings under water stress (Figure 3A). Our findings inferred that GABA-mediated tolerance might be associated with enhanced soluble sugar accumulation and osmotic adjustment for seeds germination of white clover under water stress.

Plants have evolved many important strategies including enzymatic and non-enzymatic antioxidants involved in key antioxidant enzymes (SOD, POD and CAT) and ascorbic acid-glutathione (ASA-GSH) cycle to cope with oxidative damage (Li et al., 2017; Sheteiwy et al., 2017; Jin et al., 2019; Tabaldi et al., 2019). The study of Bouché et al. (2003) found that one of possible physiological functions of GABA was to inhibit the accumulation of ROS in *Arabidopsis thaliana*. Many previous studies have reported that exogenous application of GABA improved the tolerance to water stress in different plant species associated with the improvement in antioxidant defense (Rezaei-Chiyaneh et al., 2018; Li et al., 2018a, 2019b). The PEG-induced ROS damage and membrane lipids peroxidation could be significantly alleviated by the Na^+^ or spermidine priming during seeds germination, which was related with the improvements of SOD, POD, and APX activities in seedlings of white clover under water stress (Li et al., 2014; Cao et al., 2018). It has been proved that white clover seeds pretreated with the GABA exhibited significantly lower ROS level and higher SOD, POD, CAT, APX, and MDHR activities than the seeds without GABA priming during germination under salt stress (Cheng et al., 2018). ASA and GSH are two major non-enzymatic antioxidants related to plant growth, development, stress resistance, and other biological activities. The ratios ASA/DHA and GSH/GSSG are also important indicators of the oxidation–reduction state in plant cells (Gill and Tuteja, 2010). Hydrogen sulfide (H_2_S) promoted ratios of ASA/DHA and GSH/GSSG, which contributed to better maintenance of oxidation–reduction for ROS scavenging in leaves of wheat seedlings under drought stress (Shan et al., 2018). A previous study has also demonstrated that the GABA pretreatment could significantly increase the contents of GSH, GSSG, ASA, and DHA and promoted the ratios of ASA/DHA and GSH/GSSG in leaves of creeping bentgrass under heat stress (Li et al., 2016). In addition, the GABA application directly attenuated the accumulation of H_2_O_2_ and $O_{2}^{\cdot -}$ induced by high temperature, eventually promoting seeds germination of *Arabidopsis thaliana* under heat stress (Zhang et al., 2020). Our findings demonstrated that water stress significantly increased the ROS level and MDA accumulation during germination in white clover seeds. However, the oxidative damage was significantly alleviated by the GABA priming during seeds germination, which could be associated with significant increases in activities of SOD, POD, and CAT as well as enhanced ASA-GSH cycle in seedlings under water deficient condition (Figures 5, 6).

The DREB, known as dehydration response element binding protein, is involved in comprehensive regulation of adaptive response to abiotic stresses in plants through activating downstream target genes such as *DHNs*, *rd29A*, and *COR15A* (Baker et al., 1994; Sakuma et al., 2002; Gilmour et al., 2004). Tolerance of transgenic plants with elevated levels of *DREB/CBF* was enhanced as a result of activation of DHNs encoding genes (Jaglo-Ottosen et al., 1998; Kasuga et al., 2004; Lee et al., 2005; Kobayashi et al., 2008). A *DREB2* could be significantly induced by abiotic stresses, such as drought, cold, and salt stress in wheat seedlings, and the transgenic tobacco (*Nicotiana tabacum*) overexpressing a gene *DREB2* of wheat showed improved tolerance to abiotic stresses through transcriptional activation of *DHN* genes (Kobayashi et al., 2008). Sakuma et al. (2006) found that overexpression of the *DREB2A* induced the expression of *DHN* genes to enhance tolerance to drought and heat shock. In addition, transgenic soybean overexpressing a *DREB3* exhibited significantly higher SOD activity and lower oxidative damage than wild type under water stress (Wu et al., 2015). The *DREB3*-transgenitic wheat showed significantly higher survival rates and yield than wild type under water-limited condition (Shavrukov et al., 2015). It has been found that the *DREB4* and *DREB5* were drought-inducible genes in soybean, tobacco, and white clover, and the upregulation of their expressions was beneficial for drought tolerance (Juliana et al., 2015; Ma et al., 2017; Li et al., 2018b). A previous study has also shown that the overexpression of *ARAG1*, an ABA-sensitive *DREB* gene, could enhance the germination of rice seeds under drought stress (Zhao et al., 2010). Our results revealed that the GABA significantly upregulated the expression of *DREB2*, *DREB3*, *DREB4*, and *DREB5*, indicating that the GABA-induced amelioration in seeds germination under drought stress was associated with DREB pathways in white clover.

Dehydrins are the key abundant proteins produced by seeds for later-stage embryonic development or accumulated in plants under various abiotic stresses, including dehydration, high temperature, and salt stress. The accumulation of DHNs during in late stage of seed germination is an important physiological process for maintaining water balance and increasing tolerance to water stress, because DHNs also function as hydrophilic solutes and ROS scavenging, thus sustaining the flowability of cell sap and stabilizing the structure and function of proteins to avoid structural collapse of cellular components under adverse environmental conditions (Allagulova et al., 2003; Hundertmark and Hincha, 2008). Hu et al. (2010) found that the DHNs content (31 and 40 KDa) in bermudagrass (*Cynodon dactylon*) increased significantly under drought stress, which could be associated with the improvement of drought tolerance. The DHN gene *SiDHN* in Tianshan snow lotus (*Saussurea involucrata*) significantly enhanced by abiotic stress positively contributed toward the tolerance to low temperature and drought (Guo et al., 2015). It has been reported that overexpression of wheat *DHN5* enhanced the antioxidant capacity in *Arabidopsis thaliana* resulting in improved tolerance to salt and osmotic stress (Brini et al., 2007). In the current study, water stress significantly upregulated *SK2*, *Y2K*, *Y2SK*, and *Dehydrin b* expression, but decreased the abundance of the DHN (56 KDa) in seedlings of white clover. Interestingly, the GABA priming not only further upregulated stress-induced *SK2*, *Y2K*, *Y2SK*, and *Dehydrin b* expression, but also maintained higher DHN (56 KDa) accumulation during seeds germination under water stress (Figures 7, 8). These findings suggested that the maintenance of higher *DHNs* genes expression and protein accumulation could be one of the most important survival strategies regulated by the GABA in white clover during seeds germination.

In conclusion, water stress significantly inhibited seeds germination of white clover, but seeds priming with GABA (2μmol/l) effectively alleviated the stress-induced the inhibition of seeds germination. The GABA priming effectively increased the accumulation of soluble sugars associated with significant reduction in OP under water stress. Moreover, GABA pretreatment substantially reduced the oxidative damage through enhancing enzymes (SOD, POD, CAT, APX, DHAR, GR, and MDHR) activities and contents of non-enzymatic antioxidants (ASA, DAH, GSH, and GSSG), which were involved in ASA-GSH cycle during seeds germination under water stress. In addition, the GABA-induced stress tolerance and improved seeds germination could be related to the accumulation of DHNs (56 KDa) and the higher expression of genes encoding DHNs (*SK2*, *Y2K*, *Y2SK*, and *Dehydrin b*), and transcription factors (*DREB2*, *DREB3*, *DREB4*, and *DREB5*) during seeds germination. In this study, the GABA could act as an important signaling molecule to regulate various physiological and biochemical responses to water stress during seed germination. However, metabolic functions of GABA such as regulation of GABA shunt pathway for supplying energy and carbon skeletons along with avoiding ROS accumulation, polyamine pathways, or other metabolic pathways deserves to be further studied during seeds germination in our future works.

## Data Availability Statement

The datasets presented in this study can be found in online repositories. The names of the repository/repositories and accession number(s) can be found in the article/supplementary material.

## Author Contributions

ZL conceived, designed the research, and provided different chemical reagents and experimental material. MZ conducted the experiments, evaluated the data, and completed the manuscript writing. ZL, MH, YP, LL, WL, and YZ reviewed and edited the manuscript. All authors contributed to the article and approved the submitted version.

## Conflict of Interest

The authors declare that the research was conducted in the absence of any commercial or financial relationships that could be construed as a potential conflict of interest.

## Publisher’s Note

All claims expressed in this article are solely those of the authors and do not necessarily represent those of their affiliated organizations, or those of the publisher, the editors and the reviewers. Any product that may be evaluated in this article, or claim that may be made by its manufacturer, is not guaranteed or endorsed by the publisher.

## References

[ref1] Abdul Q. Abdul R. Muhammad A. Jenks M. A. (2011). Water stress causes differential effects on germination indices, total soluble sugar and proline content in wheat (*Triticum aestivum* L.) genotypes. Afr. J. Biotechnol. 10, 14038–14045. doi: 10.5897/AJB11.2220

[ref2] Allagulova C. R. Gimalov F. R. Shakirova F. M. Vakhitov V. A. (2003). The plant dehydrins: structure and putative functions. Biochem. Biokhimiia 68, 945–951. doi: 10.1023/A:1026077825584, PMID: 14606934

[ref3] Arumingtyas E. L. Savitri E. S. Purwoningrahayu R. D. (2013). Protein profiles and dehydrin accumulation in some soybean varieties (*Glycine max* L. Merr) in drought stress conditions. American. J. Plant Sci. 4, 134–141. doi: 10.4236/ajps.2013.41018

[ref4] Baker S. S. Wilhelm K. S. Thomashow M. F. (1994). The 5′-region of *Arabidopsis thaliana corl5a* has cis-acting elements that confer cold-, drought-and ABA-regulated gene expression. Plant Mol. Biol. 24, 701–713. doi: 10.1007/BF00029852, PMID: 8193295

[ref5] Baldwin D. Crane V. Rice D. (1999). A comparison of gel-based, nylon filter and microarray techniques to detect differential RNA expression in plants. Curr. Opin. Plant Biol. 2, 96–103. doi: 10.1016/S1369-5266(99)80020-X, PMID: 10322196

[ref6] Battaglia M. Olvera-Carrillo Y. Garciarrubio A. Campos F. Covarrubias A. A. (2008). The enigmatic LEA proteins and other hydrophilins. Plant Physiol. 148, 6–24. doi: 10.1104/pp.108.120725, PMID: 18772351 PMC2528095

[ref7] Blum A. (1989). Osmotic adjustment and growth of barley genotypes under drought stress. Crop Sci. 29, 230–233. doi: 10.2135/cropsci1989.0011183X002900010052x

[ref8] Blum A. Ebercon A. (1981). Cell membrane stability as a measure of drought and heat tolerance in wheat. Crop Sci. 21, 43–47. doi: 10.2135/cropsci1981.0011183X002100010013x

[ref9] Bouché N. Fait A. Bouchez D. Møller S. G. Fromm H. (2003). Mitochondrial succinic-semialdehyde dehydrogenase of the γ-aminobutyrate shunt is required to restrict levels of reactive oxygen intermediates in plants. Proc. Natl. Acad. Sci. 100, 6843–6848. doi: 10.1073/pnas.1037532100, PMID: 12740438 PMC164534

[ref10] Bradford M. M. (1976). A rapid and senstive method for the quantitation of microgram quantities of protein untilizing the principle of protein-dye binding. Anal. Biochem. 72, 248–254. doi: 10.1016/0003-2697(76)90527-3 942051

[ref11] Brini F. Hanin M. Lumbreras V. Amara I. Khoudi H. Hassairi A.. (2007). Overexpression of wheat dehydrin *DHN-5* enhances tolerance to salt and osmotic stress in Arabidopsis thaliana. Plant Cell Rep. 26, 2017–2026. doi: 10.1007/s00299-007-0412-x, PMID: 17641860

[ref12] Cakmak I. Strbac D. Marschner H. (1993). Activities of hydrogen peroxide–scavenging enzymes in germinating wheat seeds. J. Exp. Bot. 44, 127–132. doi: 10.1093/jxb/44.1.127

[ref13] Cao Y. Liang L. Cheng B. Dong Y. Wei J. Tian X.. (2018). Pretreatment with NaCl promotes the seed germination of white clover by affecting endogenous phytohormones, metabolic regulation, and dehydrin-encoded genes Eexpression under water stress. Int. J. Mol. Sci. 19, 3570–3585. doi: 10.3390/ijms19113570, PMID: 30424572 PMC6274820

[ref14] Chance B. Maehly A. C. (1955). Assay of catalase and peroxidase. Methods Enzymol. 2, 764–775. doi: 10.1016/S0076-6879(55)02300-8

[ref15] Cheng B. Li Z. Liang L. Cao Y. Zeng W. Zhang X.. (2018). The γ-Aminobutyric acid (GABA) alleviates salt stress damage during seeds germination of white clover associated with Na^+^/K^+^ transportation, dehydrins accumulation, and stress-related genes expression in white clover. Int. J. Mol. Sci. 19:2520. doi: 10.3390/ijms19092520, PMID: 30149642 PMC6163210

[ref16] Dhindsa R. S. Plumb-Dhindsa P. Thorpe T. A. (1981). Leaf senescence: correlated with increased levels of membrane permeability and lipid peroxidation, and decreased levels of superoxide dismutase and catalase. J. Exp. Bot. 32, 93–101. doi: 10.1093/jxb/32.1.93

[ref17] Dure L. I. I. I. (1992). A repeating 11-mer amino acid motif and plant desiccation. Plant J. 3, 363–369. doi: 10.1046/j.1365-313X.1993.t01-19-00999.x 8220448

[ref18] Elstner E. F. Heupel A. (1976). Inhibition of nitrite formation from hydroxylammonium chloride: A simple assay for superoxide dismutase. Anal. Biochem. 70, 616–620. doi: 10.1016/0003-2697(76)90488-7, PMID: 817618

[ref19] Fan L. Q. (2012). Effects of γ-Aminobutyric Acid on Polyamine Metabolism and Comparative Proteomics Analysis of Melon Seedlings Under Hypoxia. Baoding: Hebei Agricultural University.

[ref20] Gao H. B. Zhang T. J. Wu X. L. Zhang G. H. Li C. R. (2007). Effect of exogenous γ-aminobutyric acid on growth and physiological metabolism in cucumber (*cucumis sativus* L.) seedlings under flooding stress. J. Inner Mongolia Agric. Univ. (Nat. Sci. Ed.) 28, 158–162. doi: 10.3969/j.issn.1009-3575.2007.03.038

[ref21] Gill S. S. Tuteja N. (2010). Reactive oxygen species and antioxidant machinery in abiotic stress tolerance in crop plants. Plant Physiol. Biochem. 48, 909–930. doi: 10.1016/j.plaphy.2010.08.016 20870416

[ref22] Gilmour S. J. Fowler S. G. Thomashow M. F. (2004). Arabidopsis transcriptional activators CBF1, CBF2, and CBF3 have matching functional activities. Plant Mol. Biol. 54, 767–781. doi: 10.1023/B:PLAN.0000040902.06881.d4, PMID: 15356394

[ref23] Guo X. Zhang L. Zhu J. Liu H. Wang A. (2015). Cloning and characterization of *SiDHN*, a novel dehydrin gene from Saussurea involucrata Kar. et Kir. that enhances cold and drought tolerance in tobacco. Plant Sci. 256, 160–169. doi: 10.1016/j.plantsci.2016.12.007 28167030

[ref24] Guo-Xing S. U. Dong B. H. Liu Y. L. Wang C. C. (2003). Metabolism and functions of γ-aminobutyric acid in higher plants. Plant Physiol. Commun. 39, 670–676. doi: 10.13592/j.cnki.ppj.2003.06.048

[ref25] Hara M. (2009). The multifunctionality of dehydrins. An Over-view. Plant Signaling Behav. 5, 503–508. doi: 10.4161/psb.11085 PMC7080494 20139737

[ref26] Harris D. Pathan A. K. Gothkar P. Joshi A. Chivasa W. Nyamudeza P. (2001). On-farm seed priming: using participatory methods to revive and refine a key technology. Agric. Syst. 69, 151–164. doi: 10.1016/S0308-521X(01)00023-3

[ref27] He F. Shen H. Q. Lin C. Fu H. Sheteiwy M. S. Guan Y. J.. (2017). Transcriptome analysis of chilling-imbibed embryo revealed membrane recovery related genes in maize. Front. Plant Sci. 7:1978. doi: 10.3389/fpls.2016.01978 28101090 PMC5209358

[ref28] Houde M. Daniel C. Lachapelle M. Allard F. Laliberté S. Sarhan F. (1995). Immunolocalization of freezing-tolerance-associated proteins in the cytoplasm and nucleoplasm of wheat crown tissues. Plant J. 8, 583–593. doi: 10.1046/j.1365-313X.1995.8040583.x, PMID: 7496403

[ref29] Hu L. Wang Z. Du H. Huang B. (2010). Differential accumulation of dehydrins in response to water stress for hybrid and common bermudagrass genotypes differing in drought tolerance plant. J. Plant Physiol. 167, 103–109. doi: 10.1016/j.jplph.2009.07.008 19716198

[ref30] Huang J. (2016). Effects of exogenous GABA on growth of cucumber seedlings under high temperature stress. J. Changjiang Veg. 8, 73–78. doi: 10.3865/j.issn.1001-3547.2016.08.031

[ref31] Hundertmark M. Hincha D. K. (2008). LEA (late embryogenesis abundant) proteins and their encoding genes in Arabidopsis thaliana. BMC Genomics 9:118. doi: 10.1186/1471-2164-9-118, PMID: 18318901 PMC2292704

[ref32] Jaglo-Ottosen K. R. Gilmour S. J. Zarka D. G. Schabenberger O. Thomashow M. F. (1998). Arabidopsis *CBF1* overexpression induces *COR* genes and enhances freezing tolerance. Science 280, 104–106. doi: 10.1126/science.280.5360.104, PMID: 9525853

[ref33] Jin X. Tao L. Xu J. Gao Z. Hu X. (2019). Exogenous gaba enhances muskmelon tolerance to salinity-alkalinity stress by regulating redox balance and chlorophyll biosynthesis. BMC Plant Biol. 19:48. doi: 10.1186/s12870-019-1660-y, PMID: 30709373 PMC6359809

[ref34] Juliana M. G. Aparecida R. F. Renata F. P. Jonas N. T. Rafaela R. R. Renato B. F. J.. (2015). Transcriptome-wide identification of reference genes for expression analysis of soybean responses to drought stress along the day. PLoS One 10:e0139051. doi: 10.1371/journal.pone.0139051 26407065 PMC4583485

[ref35] Kasuga M. Miura S. Shinozaki K. Yamaguchi-Shinozaki K. (2004). A combination of the Arabidopsis DREB1A gene and stress-inducible *rd29A* promoter improved drought- and low- temperature stress tolerance in tobacco by gene transfer. Plant Cell Physiol. 45, 346–350. doi: 10.1093/pcp/pch037, PMID: 15047884

[ref36] Khan M. I. R. Syed U. J. Priyanka C. Himanshu C. Antonio F. Nafees A. K.. (2021). Role of GABA in plant growth, development and senescence. Plant Gene 26:100283. doi: 10.1016/j.plgene.2021.100283

[ref37] Kinnersley A. M. Turano F. J. (2000). Gamma aminobutyric acid (GABA) and plant responses to stress. Crit. Rev. Plant Sci. 19, 479–509. doi: 10.1080/07352680091139277

[ref38] Kobayashi F. Ishibashi M. Takumi S. (2008). Transcriptional activation of *Cor/Lea* genes and increase in abiotic stress tolerance through expression of a wheat *DREB2* homolog in transgenic tobacco. Transgenic Res. 17, 755–767. doi: 10.1007/s11248-007-9158-z, PMID: 18034365

[ref39] Krishnan S. Laskowski K. Shukla V. Emily B. M. (2013). Mitigation of drought stress damage by exogenous application of a non-protein amino acid γ-aminobutyric acid on perennial ryegrass. Am. Soc. Horticult. Sci. 138, 358–366. doi: 10.21273/JASHS.138.5.358

[ref40] Lee S. C. Lee M. Y. Kim S. J. Jun S. H. An G. Kim S. R. (2005). Characterization of an abiotic stress-inducible dehydrin gene, *OsDhn1*, in rice (*Oryza sativa* L.). Mol Cells 19, 212–218. 15879704

[ref42] Li L. Dou N. Zhang H. Wu C. (2021). The versatile gaba in plants. Plant Signal. Behav. 16:1862565. doi: 10.1080/15592324.2020.1862565, PMID: 33404284 PMC7889023

[ref43] Li Z. Huang T. Tang M. Cheng B. Zhang X. (2019a). iTRAQ-based proteomics reveals key role of γ-aminobutyric acid (GABA) in regulating drought tolerance in perennial creeping bentgrass (*Agrostis stolonifera*). Plant Physiol. Biochem. 145, 216–226. doi: 10.1016/j.plaphy.2019.10.018, PMID: 31707249

[ref44] Li Z. Jing J. Y. Peng Y. Huang B. (2016). Metabolic pathways regulated by γ-aminobutyric acid (GABA) contributing to heat tolerance in creeping bentgrass (*Agrostis stolonifera*). Sci. Rep. 6:30338. doi: 10.1038/srep30338, PMID: 27455877 PMC4960583

[ref45] Li Z. Peng Y. Huang B. (2018a). Alteration of transcripts of stress-protective genes and transcriptional factors by γ-aminobutyric acid (GABA) associated with improved heat and drought tolerance in creeping bentgrass (*Agrostis stolonifera*). Int. J. Mol. Sci. 19, 1623–1640. doi: 10.3390/ijms19061623, PMID: 29857479 PMC6032419

[ref46] Li Z. Peng Y. Zhang J. Y. Ma X. (2012). Study on the relationship between morphological variation and geographic origin for white clover. Pratacultural Sci. 29, 1706–1714.

[ref47] Li Z. Peng Y. Zhang X. Q. Xiao M. Huang L. K. Yan Y. H. (2014). Exogenous spermidine improves seed germination of white clover under water stress via involvement in starch metabolism, antioxidant defenses and relevant gene expression. Molecules 19, 18003–18024. doi: 10.3390/molecules191118003, PMID: 25379640 PMC6271027

[ref48] Li H. C. Qiu Z. J. (2003). Summary of research on drought resistance of trees and drought resistant afforestation technology. World For. Res. 16, 17–22. doi: 10.13348/j.cnki.sjlyyj.2003.04.004

[ref49] Li Z. Wen J. Peng Y. Zhang X. Q. Ma X. Huang L. K.. (2015). Spermine alleviates drought stress in white clover with different resistance by influencing carbohydrate metabolism and dehydrins synthesis. PLoS One 10:e0120708. doi: 10.1371/journal.pone.0120708, PMID: 25835290 PMC4383584

[ref50] Li Z. Xu J. G. Gao Y. Wang C. Guo G. Y. Luo Y.. (2017). The synergistic priming effect of exogenous salicylic acid and H_2_O_2_ on chilling tolerance enhancement during maize (*Zea mays* L.) seed germination. Front. Plant Sci. 8:1153. doi: 10.3389/fpls.2017.01153 28725229 PMC5496956

[ref51] Li Z. Yong B. Cheng B. Wu X. Zhang Y. Zhang X.. (2019b). Nitric oxide, γ-aminobutyric acid, and mannose pretreatment influence metabolic profiles in white clover under water stress. J. Integr. Plant Biol. 61, 1255–1273. doi: 10.1111/jipb.12770, PMID: 30609265

[ref52] Li Z. Zeng W. Cheng B. Huang T. Zhang X. (2020). γ-Aminobutyric acid enhances heat tolerance associated with the change of proteomic profiling in creeping bentgrass. Molecules 25:4270. doi: 10.3390/molecules25184270, PMID: 32961841 PMC7571209

[ref53] Li Z. Zhu Y. He X. Yong B. Peng Y. Zhang X.. (2018b). The hydrogen sulfide, a downstream signaling molecule of hydrogen peroxide and nitric oxide, involves spermidine-regulated transcription factors and antioxidant defense in white clover in response to dehydration. Env. Exp. Bot. 161, 255–264. doi: 10.1016/j.envexpbot.2018.06.036

[ref54] Liu Y. Xu H. Wen X. X. Liao Y. C. (2016). Effect of polyamine on seed germination of wheat under drought stress is related to changes in hormones and carbohydrates. J. Integrantive Agric. 15, 2759–2774. doi: 10.1016/S2095-3119(16)61366-7

[ref55] Lyons J. M. Raison J. K. (1970). Oxidative activity of mitochondria isolated from plant tissue sensitive and resistant to chilling injury. Plant Physiol. 45, 386–389. doi: 10.1104/pp.45.4.386, PMID: 5427108 PMC396419

[ref56] Ma X. Zhang B. Liu C. Tong B. Guan T. Xia D. (2017). Expression of a populus histone deacetylase gene 84KHDA903 in tobacco enhances drought tolerance. Plant Sci. 265, 1–11. doi: 10.1016/j.plantsci.2017.09.008, PMID: 29223330

[ref57] Maria V. H. Irene R. Escribano M. I. Carmen M. Sanchez-Ballesta M. T. (2017). Deciphering the role of CBF/DREB transcription factors and dehydrins in maintaining the quality of table grapes cv. autumn royal treated with high CO_2_ levels and stored at 0°C. Front. Plant Sci. 8:1591. doi: 10.3389/fpls.2017.01591 28970842 PMC5609105

[ref58] Morgan J. M. (1984). Osmoregulation and water stress in higher plants. Annu. Rev. Plant Physiol. 35, 299–319. doi: 10.1146/annurev.pp.35.060184.001503

[ref59] Na Z. Bing Z. Hai-Jun Z. Sarah W. Chen Y. Zi-Cai Y.. (2012). Melatonin promotes water-stress tolerance, lateral root formation, and seed germination in cucumber (*Cucumis sativus* L.). J. Pineal Res. 54, 15–23. doi: 10.1111/j.1600-079X.2012.01015.x 22747917

[ref60] Nakano Y. Asada K. (1981). Hydrogen peroxide is scavenged by ascorbate-specific peroxidase in spinach chloroplasts. Plant Cell Physiol. 22, 867–880.

[ref61] Okçu G. Kaya M. D. Atak M. (2005). Effects of salt and drought stresses on germination and seedling growth of pea (*Pisum sativum* L.). Turk. J. Agric. For. 29, 237–242.

[ref62] Rezaei-Chiyaneh E. Seyyedi S. M. Ebrahimian E. Moghaddam S. S. Damalas C. A. (2018). Exogenous application of gamma-aminobutyric acid (GABA) alleviates the effect of water deficit stress in black cumin *(Nigella sativa* L.). Ind. Crop. Prod. 112, 741–748. doi: 10.1016/j.indcrop.2017.12.067

[ref63] Rhodes D. Handa S. Bressan R. A. (1986). Metabolic changes associated with adaptation of plant cells to water stress. Plant Physiol. 82, 890–903. doi: 10.1104/pp.82.4.890, PMID: 16665163 PMC1056230

[ref64] Ries S. K. (1977). Superoxide dismutases: I. Occurrence in higher plants. Plant Physiol. 59, 309–314. doi: 10.1104/pp.59.2.309 16659839 PMC542387

[ref65] Roberts J. K. DeSimone N. A. Lingle W. L. Dure L. III. (1993). Cellular concentrations and uniformity of cell-type accumulation of two lea proteins in cotton embryos. Plant Cell 5, 769–780. doi: 10.2307/3869614, PMID: 12271086 PMC160315

[ref66] Sakuma Y. Liu Q. Dubouzet J. G. Abe H. Shinozaki K. Yamaguchi-Shinozaki K. (2002). DNA-binding specificity of the ERF/AP2 domain of Arabidopsis DREBs, transcription factors involvedin dehydration-and cold-inducible gene expression. Biochem. Biophys. Res. Commun. 290, 998–1009. doi: 10.1006/bbrc.2001.6299, PMID: 11798174

[ref67] Sakuma Y. Maruyama K. Osakabe Y. Qin F. Seki M. Shinozaki K.. (2006). Functional analysis of an *Arabidopsis* transcription factor, DREB2A, involved in drought-responsive gene expression. Plant Cell 18, 1292–1309. doi: 10.1105/tpc.105.035881, PMID: 16617101 PMC1456870

[ref68] Shan C. Zhang S. Ou X. (2018). The roles of H_2_S and H_2_O_2_ in regulating ASA-GSH cycle in the leaves of wheat seedlings under drought stress. Protoplasma 255, 1257–1262. doi: 10.1007/s00709-018-1213-5, PMID: 29372337

[ref69] Shavrukov Y. Baho M. Lopato S. Langridge P. (2015). The *TaDREB3* transgene transferred by conventional crossings to different genetic backgrounds of bread wheat improves drought tolerance. Plant Biotechnol. J. 14, 313–322. doi: 10.1111/pbi.12385, PMID: 25940960 PMC11388854

[ref70] Sheteiwy M. S. Gong D. T. Gao Y. Pan R. H. Hu J. Guan Y. J. (2018). Priming with methyl jasmonate alleviates polyethylene glycol-induced osmotic stress in rice seeds by regulating the seed metabolic profile. Environ. Exp. Bot. 153, 236–248. doi: 10.1016/j.envexpbot.2018.06.001

[ref71] Sheteiwy M. S. Shao H. B. Qi W. C. Hamoud Y. A. Shaghaleh H. Khan N. U.. (2019). GABA-alleviated oxidative injury induced by salinity, osmotic stress and their combination by regulating cellular and molecular signals in rice. Int. J. Mol. Sci. 20:5709. doi: 10.3390/ijms20225709, PMID: 31739540 PMC6888568

[ref72] Sheteiwy M. Shen H. Q. Xu J. G. Guan Y. J. Song W. J. Hu J. (2017). Seed polyamines metabolism induced by seed priming with spermidine and 5-aminolevulinic acid for chilling tolerance improvement in rice (*Oryza sativa* L.) seedlings. Environ. Exp. Bot. 137, 58–72. doi: 10.1016/j.envexpbot.2017.02.007

[ref73] Sincik M. Acikgoz E. (2007). Effects of white clover inclusion on turf characteristics, nitrogen fixation, and nitrogen transfer from white clover to grass species in turf mixtures. Commun. Soil Sci. Plant Anal. 38, 1861–1877. doi: 10.1080/00103620701435621

[ref74] Sun J. K. Zhang W. H. Zhang J. M. Liu B. Y. Liu X. C. (2006). Response to droughty stresses and drought-resistances evaluation of four species during seed germination. Acta Botan. Boreali-Occiden. Sin. 26, 1811–1818. doi: 10.3321/j.issn:1000-4025.2006.09.010

[ref75] Tabaldi L. A. Cargnelutti D. Goncalves J. F. Pereira L. B. Castro G. Y. Maldaner J.. (2019). Oxidative stress is an early symptom triggered by aluminum in Al-sensitive potato plantlets. Chemosphere 76, 1402–1409. doi: 10.1016/j.chemosphere.2009.06.011 19570563

[ref76] Tang M. Y. Li Z. Luo L. Cheng B. Peng Y. (2020). Nitric oxide signal, nitrogen metabolism, and water balance affected by γ-aminobutyric acid (GABA) in relation to enhanced tolerance to water stress in creeping bentgrass. Int. J. Mol. Sci. 21:7460. doi: 10.3390/ijms21207460, PMID: 33050389 PMC7589152

[ref77] Uchida A. Andre T. I. Takashi H. (2002). Effects of hydrogen peroxide and nitricoxideon both salt and heat stress tolerance in rice. Plant Sci. 163, 515–523. doi: 10.1016/S0168-9452(02)00159-0

[ref78] Wallace W. Secor J. Schrader L. E. (1984). Rapid accumulation of γ-aminobutyric acid and alanine in soybean leaves in response to an abrupt transfer to lower temperature, drakness or chanical manipulation. Plant Physiol. 75, 170–175. doi: 10.1104/pp.75.1.170, PMID: 16663565 PMC1066856

[ref79] Wang W. B. Kim Y. H. Lee H. S. Kim K. Y. Deng X. P. Kwak S. S. (2009). Analysis of antioxidant enzyme activity during germination of alfalfa under salt and drought stresses. Plant Physiol. Biochem. 47, 570–577. doi: 10.1016/j.plaphy.2009.02.009, PMID: 19318268

[ref80] Wang G. Q. Wang X. Q. WU, B., Lu, Q. (2012). Desertification and its mitigation strategy in China. J. Resour. Ecol. 3, 97–104. doi: 10.5814/j.issn.1674-764x.2012.02.001

[ref82] Wu J. Zhuo C. Wei D. Liu H. University, N. A (2015). Physiological responses of transgenic *DREB3* drought-resistant soybeans to water stress. Crops 3, 87–92. doi: 10.16035/j.issn.1001-7283.2015.03.017

[ref83] Yong B. Xie H. Li Z. Li Y. P. Zhang Y. Nie G.. (2017). Exogenous application of GABA improves PEG-induced drought tolerance positively associated with GABA-shunt, polyamines, and proline metabolism in white clover. Plant Physiol. 8:1107. doi: 10.3389/fphys.2017.01107 PMC5744439 29312009

[ref85] Zhang Q. He D. Ying S. Lu S. Wei J. Li P. (2020). GABA enhances thermotolerance of seeds germination by attenuating the ROS damage in arabidopsis. Phyton 89, 619–631. doi: 10.32604/phyton.2020.010379

[ref86] Zhao L. Hu Y. Kang C. Tai W. (2010). *ARAG1*, an ABA-responsive DREB gene, plays a role in seed germination and drought tolerance of rice. Ann. Bot. 105, 401–409. doi: 10.1093/aob/mcp303, PMID: 20100696 PMC2826253

[ref87] Zhu J. Li Z. Kang H. Fan Y. (2006). Effects of polyethylene glycol (PEG)-simulated drought stress on Pinus sylvestris var. mongolica seed germination on sandy land. Japn. Forest Soc. Springer 11, 319–328. doi: 10.1007/s10310-006-0214-y 16110648

